# A profile of adult acute admissions to Lentegeur Psychiatric Hospital, South Africa

**DOI:** 10.4102/sajpsychiatry.v25i0.1244

**Published:** 2019-09-30

**Authors:** Herman Franken, John Parker, Robin Allen, Robert A. Wicomb

**Affiliations:** 1 Department of Psychiatry, Faculty of Medicine and Health Sciences, Stellenbosch University, Cape Town, South Africa; 2 Department of Psychiatry and Mental Health, University of Cape Town, Cape Town, South Africa; 3 Lentegeur Psychiatric Hospital, Cape Town, South Africa

**Keywords:** acute adult admissions, patient profile, substance use, violence, psychosis

## Abstract

**Background:**

The Western Cape province has the highest documented lifetime prevalence of common mental disorders in South Africa. To ensure the efficient, equitable and effective distribution of current resources, there is a need to determine the profile of patients requiring psychiatric admission.

**Aim:**

To describe patients admitted to the acute adult admissions unit at Lentegeur Hospital.

**Setting:**

Lentegeur Psychiatric Hospital is situated in Mitchells Plain, Cape Town, and serves about 1 million people from nearby urban and rural areas.

**Methods:**

This retrospective study involved an audit of all patients (18–60 years of age) admitted between 01 January 2016 and 30 June 2016. The clinical records of 573 adult patients were examined.

**Results:**

The median age of the cohort was 29 years. Most patients (63%) were educated to the secondary level. Only 12% of the patients were employed, and 37% received disability grants. More than 90% of the patients presented with psychotic symptoms. Of these, 28% presented with a first-episode of psychosis. Of all patients, 20% were referred with manic symptoms and 7% with depressive symptoms. Many patients (62%) used substances concurrently in the period leading up to admission. Significantly more males (73%) used substances compared to females (38%). Cannabis was the most widely used substance (51%), followed by methamphetamine (36%). Recent violent behaviour contributed to 37% of the current admissions. A total of 70 patients (13%) tested positive for human immunodeficiency virus (HIV), and 49 (9%) tested positive for syphilis.

**Conclusion:**

Substance use and a history of violence contributed to admissions in this population.

## Introduction

The Western Cape province has the highest documented lifetime prevalence of common mental disorders in South Africa.^1^ Lentegeur Psychiatric Hospital (LGH) is the largest of four specialist mental health hospitals in the Western Cape and serves a population of approximately 1 million people.^2^ The catchment area of the hospital includes two urban townships, Mitchells Plain and Khayelitsha; both of which are characterised by high levels of crime and substance use, and poor socio-economic status.^3,4^ Recent data confirm that Mitchells Plain and Khayelitsha have one of the highest rates of contact crimes in the province (SAPS).^5^

Methamphetamines (35%) and cannabis (22%) are the most commonly abused substances in the Western Cape.^6^ A study conducted at Stikland Psychiatric Hospital, also located in Cape Town, suggested that patients with a comorbid substance-use disorder, especially those using cannabis and methamphetamine, were significantly more likely to have violence contributing to their admission.^5^ This previous study also showed that violence and substance abuse were associated with poorer patient outcomes.

Crime, substance use and low socio-economic status are known to have an adverse impact on mental health.^7,8^ This may be expected to increase the prevalence of mental disorders in this setting and could contribute to greater pressure on health services, especially at LGH. Unfortunately, studies which describe the sociodemographic characteristics of psychiatric patients in an acute inpatient setting in South Africa are not readily available. Another concern is that there is currently no systematic or coordinated intervention strategy for managing violent patients within the South African health system. This could potentially increase the risk of patient-on-patient and patient-on-staff violence.^9,10^

Thus, for the improvement of mental health services and for the efficient, equitable and effective distribution of current resources, a better understanding of the profile of patients requiring psychiatric admission needs to be ascertained. Therefore, the aim of this study was to establish the profile of patients admitted to the acute adult admissions unit at LGH.

## Methodology

### Study design

This study was a retrospective audit of all patients (18–60 years of age) admitted under the *Mental Health Care Act* of South Africa 2002 (for involuntary or assisted care)^11^ to the acute adult admissions unit at LGH in Cape Town, South Africa, between 01 January 2016 and 30 June 2016.

### Data collection

Data were obtained by a retrospective review of the clinical records of all patients (*N* = 573) who were admitted to Lentegeur Psychiatric Hospital during the defined study period. The records included clinical interviews with patients, collateral information gained from families and clinical notes from respective referring district hospitals. Information gathered at the first encounter was recorded for patients with multiple admissions and contact visits during the study period.

Data abstraction was undertaken by the principle investigator and included demographic information (age, gender, level of education, employment, drainage area [urban or rural], marital status, disability grant recipient), clinical information (history of substance use, violence or infectious disease) and psychiatric symptoms. Given the socio-political context, this study also focused on comparing data obtained from urban referral centres; that is, between Mitchells Plain (ethnic majority mixed race) and Khayelitsha (ethnic majority black African).

Substance use was defined as the ingestion, inhalation, or injection of illicit drugs; in particular, methamphetamine (Tik), cannabis, methaqualone (Mandrax), cocaine or heroin, as reported by either the patient, or caregiver, or if noted as observed or positively tested in the documentation from the referral hospital. Substance use also includes the use of alcohol at levels clinically deemed to amount to a use disorder. Only the use of such substances clinically deemed to be related to the onset and maintenance of the current episode was considered; that is, there had to be evidence of substance use, either at the time of current admission to LGH, or in the immediate few weeks preceding, and concurrent with the onset of the symptoms that resulted in the current admission.

The history of violence was defined as a physical assault to self or others (staff, other patients, family) and/or damage to property, from the time of onset of symptoms of the current episode, up to and including the current admission. Violence, however, excluded the ingestion of substances with the aim to self-injure. Violence predating the onset of the current episode was, therefore, excluded.

### Data analysis

Demographic and clinical/behavioural data were summarised as means (± standard deviation) and median (25th – 75th percentiles) for continuous variables, as counts (percentages) for categorical variables, using Microsoft Excel. The distribution of variables across groups (gender, violence and drainage area) was compared using *t*-test and chi-square test for continuous and categorical variables, respectively. For all analyses, *p* < 0.05 was set as the level of significance. SPSS statistical software was used for all analyses.

### Ethical considerations

The study was approved by the Health Research Ethics Committee of Stellenbosch University (S16/10/237), as well as the Western Cape Health Research Committee. A waiver of informed consent was granted for this retrospective study. All identifiable patient information was anonymised.

## Results

The demographic and clinical characteristics of 573 adult psychiatric patients who were admitted to Lentegeur Psychiatric Hospital between January 2016 and June 2016 are summarised in Table 1. The median age of the cohort was 29 years. Most patients (63%) were educated to the secondary level. Only 12% of the patients were employed, and more than a third (37%) received disability grants. Most patients (62%) used substances in the period leading up to admission. Psychosis was the predominant symptom on referral, with more than 90% of cases presenting with psychotic symptoms. Of these, more than a quarter (28%) presented with first-episode psychosis. Of all patients, 20% were referred with manic symptoms and 7% with depressive symptoms. In over a third of the patients (37%), violence had been a contributing factor to their current admission. A total of 504 (89%) patients were tested for human immunodeficiency virus (HIV), and 70 (13%) tested positive; 49 patients (9%) tested positive for syphilis.

**TABLE 1:** Patient demographics and clinical characteristics with bivariate analysis across urban and rural drainage zones and in terms of gender.

Variables	Overall			Referring centre							Gender						
				Urban			Rural			*p*	Males			Females			*p*
	*n*	%	IQR	*n*	%	IQR	*n*	%	IQR		*n*	%	IQR	*n*	%	IQR	
Population	573	-	-	459	80.0	-	114	20	-	< 0.001*	383	67.0	-	190	33.0	-	< 0.01*
Median age	29	-	24–40	29	-	25–39	29.5		24.0–43.5	0.868	28	-	24–35	33	-	26–46	< 0.01*
Male	383	67	-	315	69.0	-	68	60	-	0.076	-	-	-	-	-	-	-
**Education**	-	-	-	-	-	-	-	-	-	-	-	-	-	-	-	-	0.79
Primary (Grades 1–7)	147	26	-	114	25.0	-	33	29	-	-	98	26.0	-	49	26.0	-	
Secondary (Grades 8–12)	362	63	-	295	64.0	-	67	59	-	0.547	245	64.0	-	118	62.0	-	
Tertiary	34	6	-	25	5.0	-	9	8	-	-	20	5.0	-	14	7.0	-	
Unknown	30	5	-	25	5.0	-	5	4	-	-	20	5.0	-	10	2.0	-	
Substance use	353	62	-	287	62.5	-	66	58	-	0.390	281	73.0	-	72	38.0	-	< 0.01*
Married	64	11	-	14	12.0	-	50	11	-	0.739	30	8.0	-	34	18.0	-	< 0.01*
Employed	68	12	-	50	11.0	-	18	16	-	0.144	47	12.0	-	21	11.0	-	0.69
Disability grant	211	37	-	173	38.0	-	37	32.5	-	0.279	128	34.0	-	83	44.0	-	0.02*
Psychosis	517	90	-	411	89.5	-	105	92	-	0.487	354	92.0	-	163	85.0	-	< 0.01*
First-episode psychosis	159	28	-	122	27.0	-	37	33	-	0.198	107	28.0	-	52	27.0	-	0.92
Violence	211	37	-	167	36.5	-	44	39	-	0.745	159	41.5	-	53	28.0	-	< 0.01*
Mania	114	20	-	90	20.0	-	24	21	-	0.793	63	16.0	-	51	27.0	-	< 0.01*
Depression	41	7	-	27	6.0	-	14	12	-	0.025*	8	2.0	-	33	17.0	-	< 0.01*
Self-harm	48	8	-	36	8.0	-	12	10.5	-	0.450	32	8.0	-	16	8.0	-	1.0
Full medical	509	89	-	406	88.5	-	103	90	-	0.662	336	88.0	-	174	91.0	-	0.26
Syphilis	49	9	-	37	8.0	-	12	11	-	0.456	26	7.0	-	23	12.5	-	0.04*
HIV tested	504	89	-	410	91.0	-	94	83	-	0.027*	377	67.0	-	189	33.0	-	0.04*
HIV-positive	70	13	-	59	13.0	-	11	10	-	0.426	18	5.0	-	52	28.0	-	< 0.01*

* , Indicates statistical significance at *p* < 0.05

The majority of patients (80%) were admitted from the urban referral centres (Table 1). A significantly higher proportion of patients were tested for HIV in urban referral centres (91%) compared to rural centres (83%). There were no significant differences between urban and rural cohorts in regard to other parameters investigated.

Men represented the majority (67%) of the cohort and, compared to women, were significantly more likely to have used substances (73% vs. 38%), present with psychosis (92% vs. 85%), have violence contribute to their admission (42% vs. 28%), and be tested for HIV (67% vs. 33%) (Table 1). Women were significantly more likely to be older (mean 33 years, range 26–46 years) than males (mean 28 years, range 24–35 years) and also more likely to be married (18% vs. 8%), receive disability grants (44% vs. 34%), present with mania (27% vs. 16%), present with depression (17% vs. 2%) and test positive for HIV (28% vs. 5%) and syphilis (13% vs. 7%).

Of all patients, 234 (41%) were from Mitchells Plain, 213 (37%) were from Khayelitsha and 126 (22%) were from other rural referral sites (Table 2). Patients from Mitchells Plain (mean age 31 years, range 26–40 years) were significantly older compared to those from Khayelitsha (mean age 28 years, range 23–37.5 years). Patients from Mitchells Plain were significantly more likely to have received disability grants (46% vs. 30%) and to be married (17% vs. 5%). Patients from Khayelitsha were more likely to be referred with a first episode of psychosis compared to those from Mitchells Plain (32% vs. 21%). There were no significant differences between patients from Mitchells Plain and Khayelitsha in regard to other parameters investigated.

**TABLE 2:** Demographic and clinical characteristics of patients from Mitchells Plain and Khayelitsha.

Variables	Overall (*n* = 447)			Area						
				Mitchells Plain (*n* = 234)			Khayelitsha (*n* = 213)			*p*
	*n*	%	IQR	*n*	%	IQR	*n*	%	IQR	
Median age	29	-	25–29	31	-	26–40	28	-	23–37.5	0.02*
Male	303	68	-	149	64.0	-	154	73.0	-	0.06
**Education**	-	-	-	-	-	-	-	-	-	
Primary (Grades 1–7)	112	25	-	68	29.0	-	44	21.0	-	0.13
Secondary (Grades 8–12)	286	64	-	138	59.0	-	148	69.5	-	
Tertiary	24	5	-	13	6.0	-	11	5.0	-	
Unknown	25	6	-	15	6.0		10	5.0	-	
Substance use	276	62	-	141	60.0	-	135	63.0	-	0.56
Employed	48	11	-	30	13.0	-	18	8.5	-	0.17
Violence	166	37	-	94	40.0	-	72	34.0	-	0.20
Disability grant	170	39	-	106	46.0	-	64	30.0	-	< 0.01*
Married	50	11	-	39	17.0	-	11	5.0	-	< 0.01*
Psychosis	403	90	-	205	88.0	-	198	93.0	-	0.08
FE psychosis	118	26	-	50	21.0	-	68	32.0	-	0.01*
Mania	89	20	-	45	19.0	-	44	21.0	-	0.72
Depression	27	6	-	18	8.0	-	9	4.0	-	0.16
Self-harm	34	8	-	20	8.5	-	14	7.0	-	0.48
Full medical	396	89	-	202	86.0	-	194	91.0	-	0.136
Syphilis	35	8	-	20	9.0	-	15	7.0	-	0.60
Tested for HIV	400	91	-	205	89.5	-	195	92.0	-	0.32
HIV-positive	59	14	-	22	10.0	-	37	18.0	-	0.02*

* , Indicates statistical significance at *p* < 0.05

Violence was found to be a contributing factor to admission in 37% of the studied patients (Table 3). Significantly, more men (41.5%) were reported to present with violent behaviour compared to women (28%). Patients using substances (45%) were more likely to have been violent compared to those who did not use substances (25%). Patients who presented with depression were less likely to have been violent (22%) compared to those who were not depressed (38%). There were no significant differences in violence in regard to other parameters investigated.

**TABLE 3:** Demographic and clinical characteristics of patients where violence was reported.

Variables	Violence							
	No (*n* = 361)			Yes (*n* = 212)			*p*
	*n*	%	IQR	*n*	%	IQR	
**Median age**	30	-	25–42	28.0	-	24–34	0.09
**Gender**							
Male	224	58.5	-	159	41.5	-	< 0.01*
Female	137	72.0	-	53	28.0	-	
**Education**							
Primary (Grades 1–7)	93	63.0	-	54	37.0	-	0.54
Secondary (Grades 8–12)	226	62.0	-	136	38.0	-	
Tertiary	25	73.5	-	9	26.5	-	
Unknown	17	57.0	-	13	43.0	-	
**Substance use**							
Yes	195	55.0	-	158	45.0	-	< 0.01*
No	166	75.5	-	54	25.0	-	
**Married**							
Yes	42	66.0	-	22	34.0	-	0.68
No	319	63.0	-	190	37.0	-	
**Employed**							
Yes	48	71.0	-	20	29.0	-	0.18
No	313	62.0	-	192	38.0	-	
**Disability grant**							
Yes	128	61.0	-	82	39.0	-	0.47
No	233	64.0	-	130	36.0	-	
**Psychosis**							
Yes	319	62.0	-	197	38.0	-	0.08
No	42	74.0	-	15	26.0	-	
**First-episode psychosis**							
Yes	101	63.5	-	58	36.5	-	0.92
No	260	63.0	-	154	37.0	-	
**Mania**							
Yes	70	62.0	-	43	38.0	-	0.83
No	291	63.0	-	169	37.0	-	
**Depression**							
Yes	32	72.0	-	9	22.0	-	0.04*
No	329	62.0	-	203	38.0	-	
**Self-harm**							
Yes	26	54.0	-	22	46.0	-	0.21
No	335	64.0	-	190	36.0	-	

* , Indicates statistical significance at *p* < 0.05

Out of the 573 studied patients, the majority (62%) had used substances during the course of their current illness (Table 4). These patients were significantly younger compared to those who did not use substances (mean age 27 years, range 23–33years vs. mean age 37 years, range 28–47 years). Significantly more men (73%) used substances compared to women (38%). Tertiary-level education was associated with a lower likelihood of substance use. Violent patients, patients presenting with a first-episode psychosis and patients diagnosed with syphilis were significantly more likely to be substance users. Married patients, patients receiving a disability grant, patients referred with mania and depressed patients were significantly less likely to have used substances. There were no significant differences in substance use in regard to rural or urban domicile, being tested for HIV and self-harm.

**TABLE 4:** Demographic and clinical characteristics of patients who used substances prior to admission.

Variables	Overall (*N* = 573)			Area						
				Mitchells Plain (*n* = 220)			Khayelitsha (*n* = 353)			*p*
	*n*	%	IQR	*n*	%	IQR	*n*	%	IQR	
**Median age**	29	-	24–40	37	-	28–37	27	-	23–33	< 0.01*
**Gender**										
Male	383	100.0	-	102	27.0	-	281	73.0	-	< 0.01*
Female	190	100.0	-	118	62.0	-	72	38.0	-	
**Education**										
Primary (Grades 1–7)	147	100.0	-	60	41.0	-	87	59.0	-	< 0.01*
Secondary (Grades 8–12)	363	100.0	-	127	35.0	-	236	65.0	-	
Tertiary	34	100.0	-	22	65.0	-	12	35.0	-	
Unknown	30	100.0	-	12	40.0	-	18	60.0	-	
**District**										
Urban	459	100.0	-	172	37.5	-	287	62.5	-	0.39
Rural	114	100.0	-	48	42.0	-	66	58.0	-	
**Married**										
Yes	64	100.0	-	38	59.0	-	26	41.0	-	< 0.01*
No	509	100.0	-	182	36.0	-	327	64.0	-	
**Employed**										
Yes	68	100.0	-	27	40.0	-	41	60.0	-	0.90
No	505	100.0	-	193	38.0	-	312	62.0	-	
**Disability grant**										
Yes	211	100.0	-	105	50.0	-	106	-	-	< 0.01*
No	362	100.0	-	115	32.0	-	247	-	-	
**Psychosis**										
Yes	517	100.0	-	193	37.0	-	324	63.0	-	0.09
No	56	100.0	-	27	48.0	-	29	52.0	-	
**First-episode psychosis**										
Yes	159	100.0	-	44	28.0	-	115	72.0	-	< 0.01*
No	414	100.0	-	176	42.5	-	238	57.5	-	
**Violence**										
Yes	212	100.0	-	54	25.5	-	158	74.5	-	< 0.01*
No	361	100.0	-	166	46.0	-	195	54.0	-	
**Mania**										
Yes	114	100.0	-	57	50.0	-	57	50.0	-	< 0.01*
No	459	100.0	-	163	35.5	-	296	64.5	-	
**Depression**										
Yes	41	100.0	-	26	63.0	-	15	37.0	-	< 0.01*
No	532	100.0	-	194	36.5	-	338	63.5	-	
**Self-harm**										
Yes	48	100.0	-	17	35.0	-	31	65.0	-	< 0.01*
No	525	100.0	-	203	39.0	-	322	61.0	-	
**Full medical**										
Yes	510	100.0	-	188	37.0	-	322	63.0	-	0.03*
No	63	100.0	-	32	51.0	-	31	49.0	-	
**Syphilis**										
Yes	49	100.0	-	12	24.5	-	37	75.5	-	0.05*
No	524	100.0	-	208	40.0	-	316	60.0	-	
**HIV tested**										
Yes	505	100.0	-	192	38.0	-	313	62.0	-	0.69
No	68	100.0	-	28	41.0	-	40	59.0	-	

* , Indicates statistical significance at *p* < 0.05

Cannabis was the most widely used substance (51%), followed by methamphetamine (36%) (Table 5). Men were statistically more likely to have used methamphetamines, cannabis and methaqualone. There was no statistical difference in the use of alcohol or heroine between males and females.

**TABLE 5:** Relationship between specific substances, referral centre and gender.

Variables	Overall (*N* = 573)		Referral centre					Gender				
			Urban (*n* = 459)		Rural (*n* = 114)		*p*	Males (*n* = 383)		Females (*n* = 190)		*p*
	*n*	%	*n*	%	*n*	%		*n*	%	*n*	%	
Substance use	353	62.0	287	62.5	66	58	0.39	281	73	72	38	< 0.01*
Methamphetamine	204	36.0	168	37.0	36	32	0.33	163	43	41	22	< 0.01*
Cannabis	288	50.0	234	51.0	54	47	0.53	239	62	49	26	< 0.01*
Methaqualone	81	14.0	60	13.0	21	18	0.18	71	19	10	5	< 0.01*
Heroin	16	3.0	14	3.0	2	2	0.55	13	3	3	2	0.29
Alcohol	100	17.5	81	18.0	19	17	0.89	73	19	27	14	0.16

* , Indicates statistical significance at *p* < 0.05.

## Discussion

The aim of our study was to establish the profile of adult admissions to the acute wards at LGH. Most patients were men (67%), had a secondary level of education (63%), were unemployed (88%), had a history of concurrent substance abuse (62%) and/or concurrent violence (37%), and presented with psychotic symptoms (90%).

A previous study found that patients admitted to LGH between 01 August 2012 and 31 January 2013 were also mostly men (65.6%) and aged younger than 35 years (58%).^12^ In the current study, admissions were predominantly male, with an overall younger mean age of 29 years. In comparison, a study conducted in the UK found a majority of female admissions with an older mean age.^13^

The SACENDU data indicate high levels of substance use, especially illicit drug use, among the general population within the LGH drainage regions.^6^ Our findings mirror this pattern as 61.6% (*n* = 353) of the patients were found to have misused substances, particularly cannabis and methamphetamines. Younger age, first-episode psychosis, violence, male gender, and being unmarried and unemployed were features significantly associated with acute admissions who abused substances. Those patients who were married, educated to a tertiary level, presented with a mood disorder (mania or depression), and received a disability grant were significantly less likely to abuse substances. Comorbid substance use among psychiatric inpatients is associated with several negative outcomes, including aggression and violence, medication non-compliance, rehospitalisation, and poor social reintegration after discharge.^14,15,16,17^

Psychosis was the predominant finding in more than 90% of the cases, with a slightly higher prevalence among men. First-episode psychosis constituted only 28% of all admissions. This might suggest that the current community mental health care system is failing to maintain remission. This could be due to ill-equipped primary health care services and a lack of psychosocial rehabilitation facilities.^18^ The repeated admission of patients to specialised psychiatric hospitals due to relapse demands attention.

Notably, 37% of the overall admissions in the current study were found to have displayed violent behaviour during the period leading up to, or upon, admission. In contrast, Chaput et al. reported that only 7.4% of visits to participating psychiatric emergency services in Quebec, Canada were marked by current aggression.^19^ Furthermore, our study found the violent cohort to be significantly associated with the male gender and substance use.

Rothärmel et al. found substance use and the severity of psychotic symptoms to be significant factors associated with violent behaviour in psychotic patients.^20^ Luckhoff et al. found acutely psychotic and violent patients to be at an increased risk of requiring treatment in specialised seclusion rooms (rather than in dormitory rooms).^21^ This requires some consideration in the South African context where the management of psychotic and violent patients inside standard dormitories poses considerable risk to fellow patients, as well as to staff members. However, a reliance on specialised seclusion rooms has been associated with an increased morbidity and mortality within tertiary psychiatric centres.^21^

In terms of the 72-h observation requirements of the *Mental Health Care Act*,^11^ a higher proportion of intoxicated and violent patients may be prioritised for admission to tertiary psychiatric centres (Level 2 hospitals such as LGH). Such patients may not be able to be contained by the limited infrastructure and the smaller number of mental health clinicians (medical, nursing, security personnel) within the district centres, such as Mitchells Plain and Khayelitsha District Hospitals. The result may be a system where specialised mental health centres are disproportionately managing intoxication, whereas patients with enduring psychiatric illnesses are managed with an early down-referral to primary health clinics, which are unable to provide the full range of services. Certainly, this merits consideration and possible investigation; it is critical that available resources are utilised in a manner that ensures the delivery of optimal and cost-effective mental health services.

Combined unemployment statistics for Khayelitsha and Mitchells Plain showed an unemployment rate of 32% in 2014.^22^ The current study shows the psychiatric population to be particularly vulnerable in this regard, as only 12% of the admissions were employed. More than a third of admissions were in receipt of a disability grant, and almost a third of grant recipients misused substances. This is an obvious ethical and systemic concern because it appears government may be funding substance use among persons with mental illness. Only a small minority – who were more likely to be female – were married. This leaves the majority of psychiatric admissions to rely on parents or extended family, where available and willing, for caregiver support. The burden on those taking care of patients with a chronic psychiatric illness negatively impacts their quality of life in various areas.^23^ These factors highlight the need for greater cooperation, planning, and possibly policy generation in the Department of Social Services.

The LGH catchment area is characterised by severe socio-economic challenges. Violence and other contact crimes abound within its communities.^3,4^ Khayelitsha’s proportion of violent crimes, captured as crimes against a person (contact crimes), are considerably higher than the national average, and crime is also prevalent in Mitchells Plain.^24^ Previous studies have shown violence to be a considerable problem within South African psychiatric centres.^25,26^ Forte et al. report that hyper-vigilance and fear among staff as a result of exposure to violent behaviour negatively impact care.^27^ The issue of violence in the LGH setting could increase the medico-legal risk. Furthermore, the risk of burn-out among staff also warrants urgent consideration in this setting.^25,28^

Comorbid medical illness also requires further consideration in our psychiatric population. Although uniform testing for HIV is not included in standard operating procedures at LGH, this is advised where there is a high index of suspicion based on clinical grounds. HIV infection brings with it other concerns, such as associated medical complications and related cost of care.^29^ The prevalence of HIV infection in our population was found to be 13% and significantly higher among women admitted to LGH. The considerations around HIV testing probably deserve attention as it is unclear why significantly fewer females, compared to males, were tested during the psychiatric admission process. Syphilis is still a major consideration among psychiatric patients as 9% tested positive in our study. The disease was significantly associated with substance misuse in this cohort. The overlap of these two epidemics of sexually transmitted diseases that have the potential for neuropsychiatric sequelae is a concerning feature of the local mental health landscape.

A limitation of this retrospective study is that our data depended on the accuracy of the original source, as reliance was almost solely placed on self-reported data and collateral information. This is particularly relevant to our substance use data, as very few patients had toxicology done at referral centres. The sample period of 6 months may also limit the accuracy of data, especially regarding seasonal variance, but we believe a large cohort allows for sufficient generalisation of data. Lastly, the retrospective nature of our study limits the inference of causality.

## Conclusion

Our study highlighted the profile of patients referred to LGH and identified salient factors impacting care. In describing the profile of acute adult patient admissions to LGH, we identified substance use and a propensity for violence as significant factors influencing the likelihood of admission. These factors place strain on available resources, and complicate treatment. This study thus argues for the expansion and capacitation of mental health services particularly at the tertiary level in the Western Cape and potentially across the broader mental health platform in South Africa. As this study may be suggestive of particular medico-legal risk at both corporate and clinical governance levels at medical institutions, we invite planners within the Department of Health, as well as other stakeholders in the government, to take heed of this burgeoning crisis and implement specific strategies for addressing these problems, before the effectiveness of mental health services as a whole is further undermined.
